# Pollution and health risk assessments related to heavy metals on three prominent beaches in Makkah Province, Kingdom of Saudi Arabia: Concerning levels of cadmium pollution

**DOI:** 10.1371/journal.pone.0311189

**Published:** 2024-10-21

**Authors:** Abdulaziz Alayyafi, Mohammad Ebqa’ai, Omar Alboqai, Ahmed Abotaleb, Ahmed Eldesoky, Abeer El Shahawy

**Affiliations:** 1 Department of Chemistry, University College in Al-Qunfudah, Umm Al-Qura University, Mecca, Saudi Arabia; 2 Department of Chemistry, Physics, and Engineering, Cameron University, Lawton, Oklahoma, United States of America; 3 Department of Chemistry, Oklahoma State University, Stillwater, Oklahoma, United States of America; 4 Department of Nutrition and food Science, Faculty of Agriculture, Jerash University, Jerash, Jordan; 5 Department of Civil Engineering, Faculty of Engineering, Suez Canal University, Ismailia, Egypt; 6 Chemical Engineering Department, High Institute of Engineering & Technology, New Damietta, Egypt; University of Peshawar National Centre of Excellence in Geology, PAKISTAN

## Abstract

Beach pollution can impact the health of people living in or visiting coastal areas. The primary goal of this research was to investigate the presence of heavy metal pollutants and associated health risks in three distinct coastal cities, Jeddah, Al-Lith, and Al-Qunfudhah, located along Saudi Arabia’s Red Sea coast. Forty-three soil samples were collected from different locations, heavy metals including Fe, Zn, Mn, Cu, Cd, and Pb were isolated, and analyzed using inductively coupled plasma optical emission spectroscopy. Various metrics such as pollution indices (PI), integrated pollution indices (IPI), enrichment factors (EF), daily dose averages (ADD), hazard quotients (HQ), and hazard indices (HI), as well as principal component analysis (PCA) and cluster analysis were employed to evaluate the environmental impacts and health risks posed by these heavy metals. The results revealed that Fe, Pb, Cu, Mn, and Zn concentrations in beach zones were below their respective background levels, while concentrations of Cd exceeded corresponding background levels. PCA revealed the highest levels of Pb, Fe, and Mn on Al-Qundudah beach, while Cd and Cu were highest on Al-Lith beach, and Zn was highest on Jeddah beach. PI values highlighted extremely high levels of Cd pollution on all designated beaches. The findings provide a foundational basis for further investigations into health problems potentially related to Cd contamination, such as chronic kidney disease (CKD), osteomalacia, and osteoporosis.

## 1. Introduction

Worldwide concern over environmental contamination and its eco-toxicological impact has grown due to expanded urbanization, economic growth, human population, sewage wastewater, fossil fuel combustion, atmospheric deposition, and intensive industrial development [1, 2]. The health of coastal environments significantly impacts the commercial and economic progress of coastal regions. However, despite their importance, coastal ecosystems face the continuous release of chemical contaminants from both natural sources such as rocks and human-made activities, driven by ongoing development in these areas [3]. Heavy metals have attracted significant attention as pollutants infiltrating coastal environments due to their toxic nature, widespread presence, abundance, resistance to breakdown, and ability to accumulate in organisms [4]. Additionally, heavy metal pollution of soil presents major risks to ecosystems and human health because it can enter the body through food and skin contact, as well as other exposure routes [5]. Research indicates a common pattern for the environmental impacts of heavy metals—they tend to accumulate in the environment, posing challenges for human, plant, and animal health due to their stability. Toxic elements including heavy metals are widespread in coastal zones and can enter the human body through inhalation, ingestion, and skin contact [6–12]. Moreover, levels of heavy metals in coastal areas can serve as proxies for pollution levels in urban and industrial regions.

Scientists worldwide, and particularly in coastal cities, prioritize controlling heavy metals on beaches [13, 14]. Various studies have measured levels of heavy metals in coastal regions, including those adjacent to the Red Sea. In locations such as the Gulf of Aden, the Red Sea in Yemen and Egypt, and the Gulf of Aqaba in Jordan, measured concentrations were lower than baseline levels [13]. Conversely, substantial amounts of heavy metals have been identified in the Gulf of Mannar, India [13]. European studies have also revealed significant concentrations, such as in Espinho, Portugal, where beach sediments contained 371.4, 59.6, and 96.9 ppm of copper (Cu), lead (Pb), and zinc (Zn), respectively [14]. Although some studies have assessed heavy metal levels in Red Sea coastal areas of Saudi Arabia [14], no prior investigations of this kind have been conducted in the Al-Lith and Al-Qunfudhah areas within Makkah Province. Therefore, the present study assessed the concentrations of six metals—iron (Fe), manganese (Mn), Cu, Zn, cadmium (Cd), and Pb—by collecting 45 soil samples from beaches of these two cities, along with Jeddah beach, all situated along the Saudi Arabian coast.

The primary objectives of this study were to investigate the sources and contributions of soil-based heavy metals across the three beaches, and quantitatively assess the health risks posed by these metals to various demographic groups, including children, adult females, and adult males. Furthermore, the study aimed to analyze the health risks to vulnerable populations arising from different sources. To achieve these aims, various indices were assessed including metal enrichment factor (EF), pollution index (PI), integrated pollution index (IPI), potential human health risk hazard quotient, and hazard index (HI). These indices serve as essential tools for comprehensively evaluating concentrations of heavy metals with environmental significance. They aid in conducting a thorough assessment of pollution levels and gauging the presence and intensity of human-induced pollutant deposition in surface soil [14, 15].

## 2. Methodology

### 2.1. Sourcing and preparation

**Fig 1:**
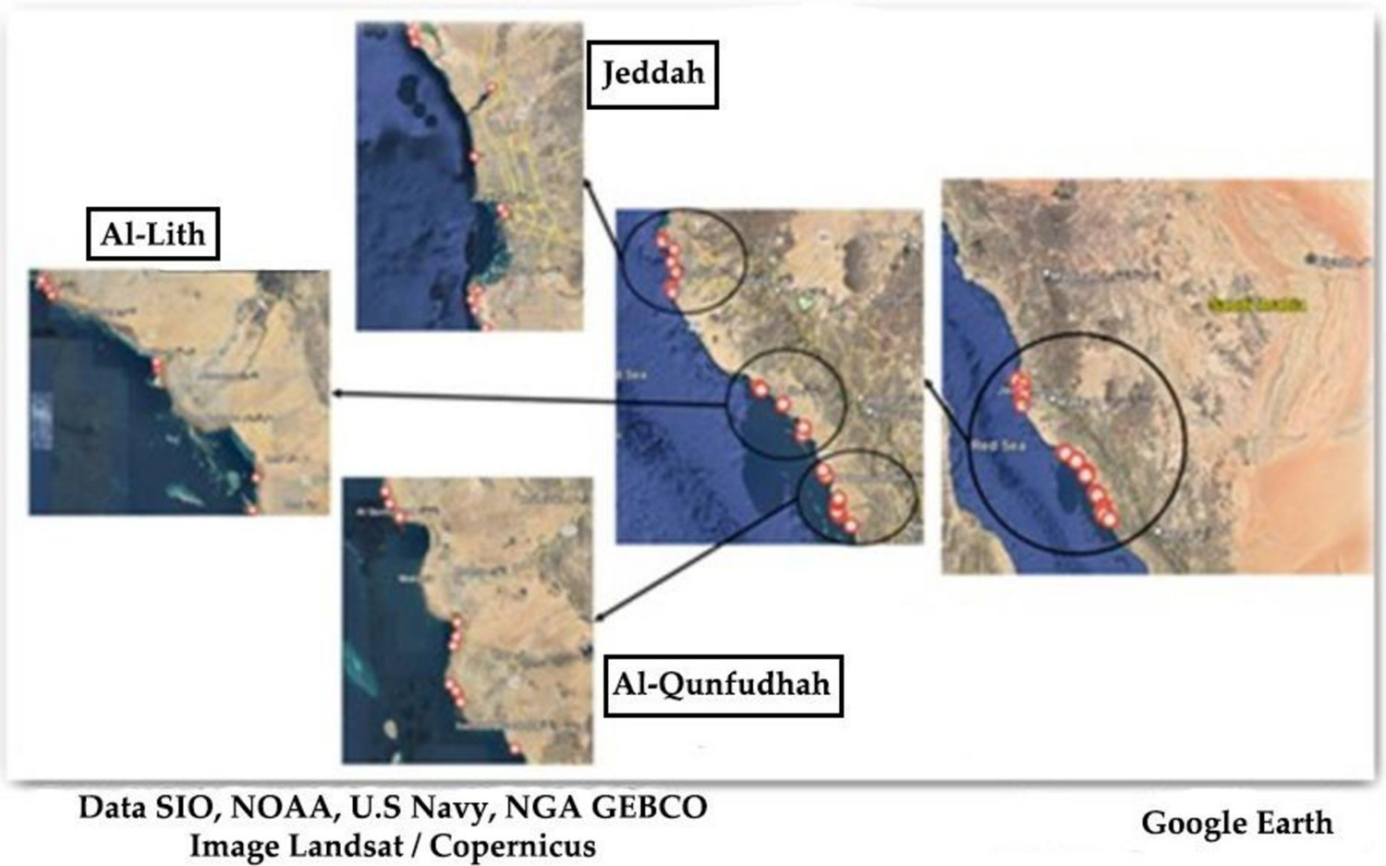
Sampling locations shown using Google Earth.

This study focused on three primary beaches within the Makkah province of Saudi Arabia. These beaches are situated in three distinct cities—Jeddah, Al-Lith, and Al-Qunfudhah—which collectively form a linear stretch along the west coast of the Red Sea. Samples were collected between June and July 2023. The land areas of these cities are approximately 1765 km^2^ for Jeddah, 1181 km^2^ for Al-Lith, and 8.2 km^2^ for Al-Qunfudhah (**Fig 1**) (S1 Table in S1 Data for raw data) [16–18].

The study focused on coastal regions to investigate the diverse sources of contamination impacting this area, known for its commercial and industrial importance. Previous research primarily centered on Jeddah, renowned for its substantial industrial development over the past two decades [18]. The Jeddah environment has been impacted by significant infrastructure, including electrical power plants, a substantial municipal sewage system, several desalination facilities, a pteropine refinery plant, and Aramco refinery facility. Al-Lith and Al-Qunfudhah, coastal cities on the southwest shore of the Red Sea, host desalination plants and power generation facilities. As human population and industrialization continue to expand in the study area, the issue of environmental pollution, including heavy metals, becomes increasingly critical. This is especially important for coastal habitats along the Red Sea in Saudi Arabia, especially saline mud flats (Sabkha), mangrove swamps, palm groves, coral reefs, and sea grass beds [19].

Heavy metals can be transported in water as dissolved species or as part of suspended sediments, eventually depositing in bottom sediments and altering their original physicochemical properties. The local human population living near this area, and potentially accessing this location, faces risks from heavy metal bioaccumulation due to the high biological activities in this environment [20].

In the laboratory, soil samples underwent several steps for preparation. They were initially dried overnight, stored in a desiccator for 24 h, sieved through a 100-mesh stainless steel sieve, transferred into plastic tubes, sealed with parafilm, and left to air-dry until analysis. All concentrations reported in this study are based on dry weight measurements [21].

### 2.2. Experiments

Deionized water with a high resistance of 18.2 MΩ (Nanopure water, Barnstead) and Aqua regia solution (consisting of 70% nitric acid and 35% hydrochloric acid) served as solvents. Stock solutions, each containing 100 micrograms per gram of the desired metals Fe, Pb, Zn, Cu, Mn, and Cd, were employed to create standard solutions. All solvents and solutions were of analytical reagent grade and procured from Merck (Darmstadt, Germany).

The study focused on three primary beaches—Jeddah, Al-lith, and Al-Qunfudhah—as the sampling sites. Forty-five samples were collected during the summer of 2023 (15 from each beach). These samples were carefully stored in labeled polyethylene containers and shielded from direct sunlight. Upon collection, the samples underwent an initial air-drying process overnight and were subsequently sieved through a 0.05-mm stainless steel sieve. For processing, 1 g of sieved sample was ashed for 30 min at 350°C. Following this, 8 ml of acid mixture (2 ml of 70% HNO_3_ and 6 ml of 35% HCl) was added and sample were incubated at room temperature for 12 h. Notably, the calcareous soil sample did not undergo any additional pre-treatment.

Digestion of samples was carried out using a Milestone MLS 1200 Mega microwave digestion instrument (Company, City, Country). Upon cooling, 2 ml of saturated boric solution was added to vials, which were then sealed and reheated in a microwave oven for 3 min at 300 W. The resulting clear solutions were carefully transferred into 50 ml flasks and brought to volume using deionized water [22]. Extraction of heavy metals, specifically Fe, Cd, Cu, Mn, Pb, and Zn, was performed in triplicate, employing a standardized method that involved sequential extraction into six operationally defined fractions. Extractions were conducted using 50 ml polypropylene centrifuge tubes. The first water-soluble fraction was obtained using 15 ml of deionized water. The precipitate was removed and analyzed for heavy metals, while the residue underwent a rinse with 8 ml of deionized water. After a 30 min centrifugation, the second precipitate was discarded. The volume of rinse water used was minimized to prevent excessive solubilization of solid materials, particularly organic matter [23].

### 2.3. Product characterization

The collective concentration of specific heavy metals (Fe, Pb, Cu, Mn, Zn, and Cd) was determined by inductively coupled plasma optical emission spectroscopy (ICP-OES) using a iCAP 7000 Plus Series instrument (Thermo Scientific, City, Country). This analysis encompassed a spectral range from 167 nm to 852 nm. Quality control measures were implemented using standard metal solutions and triplicate samples, particularly for the fraction that was soluble in water. This ensured a consistent and reliable assessment of heavy metal concentrations within samples.

## 3. Results and discussion

### 3.1. Heavy metal levels

Coastal areas of Jeddah, Al-Lith, and Al-Qunfudhah yielded notable mean, minimum, maximum, and standard deviation (SD) values for heavy metal concentrations—specifically Fe, Zn, Mn, Cu, Pb, and Cd—as detailed in **Tables 1–2.** The prevalence of these heavy metals in soil along these urban coastlines can be attributed to various human activities including transportation-related emissions from car exhausts, abrasion from tires and brakes, power generation involving combustion emissions, and the burning of fossil fuels across residential, commercial, and industrial domains [24]. Mean concentrations of heavy metals differed between beaches; Jeddah beach was ordered Fe > Zn > Mn > Cu > Pb > Cd (higher to lower), Al-Lith beach was ordered Fe > Mn > Zn > Cu > Pb > Cd, and Al-Qunfudhah beach was ordered Fe > Mn > Zn > Pb > Cu > Cd.

**Table 1:** Heavy metal concentrations (ppm) on Jeddah beach.

Element	Mean ^a^	Min ^a^	Max ^a^	SD ^a^	Background ^b^
Iron	5444	748.99	22613	5421.0	47200
Lead	10.87	3.320	19.58	4.6680	20.000
Copper	11.76	10.57	19.06	7.9260	45.000
Manganese	84.32	12.88	550.69	127.20	850.00
Zinc	197.3	35.60	650.74	221.07	95.000
Cadmium	1.720	0.450	3.4000	0.8750	0.3000

^a^ Values are based on 15 measurements (**S2 Table** in S1 Data for raw data)

^b^ World average concentrations [25, 29].

**Table 2:** Heavy metal concentrations (ppm) on Al-Lith beach.

Element	Mean ^a^	Min ^a^	Max ^a^	SD ^a^	Background ^b^
Iron	11,405	2434.9	22741	7415.2	47,200
Lead	13.044	0.9080	24.18	5.4559	20.000
Copper	21.847	4.6700	69.51	16.758	45.000
Manganese	162.30	31.740	302.71	98.069	850.00
Zinc	92.030	20.870	177.68	44.699	95.000
Cadmium	3.3861	1.6200	5.188	1.0393	0.3000

^a^ Values are based on 15 measurements (**S3 Table** in S1 Data for raw data)

^b^ World average concentrations [25, 29].

**Table 3:** Heavy metal concentrations (ppm) on Al-Qunfudhah beach.

Element	Mean ^a^	Min ^a^	Max ^a^	SD ^a^	Background ^b^
Iron	13,349	3248.32	19007	4492.0	47200
Lead	18.195	0.1600	61.97	16.074	20.000
Copper	12.107	3.7300	18.92	4.3729	45.000
Manganese	253.84	472.31	71.40	96.804	850.00
Zinc	75.614	46.010	152.98	25.005	95.000
Cadmium	2.8333	1.3900	4.940	0.9188	0.3000

^a^ Values are based on 15 measurements (**S4 Table** in S1 Data for raw data)

^b^ World average concentrations [25, 29].

**Fig 2:**
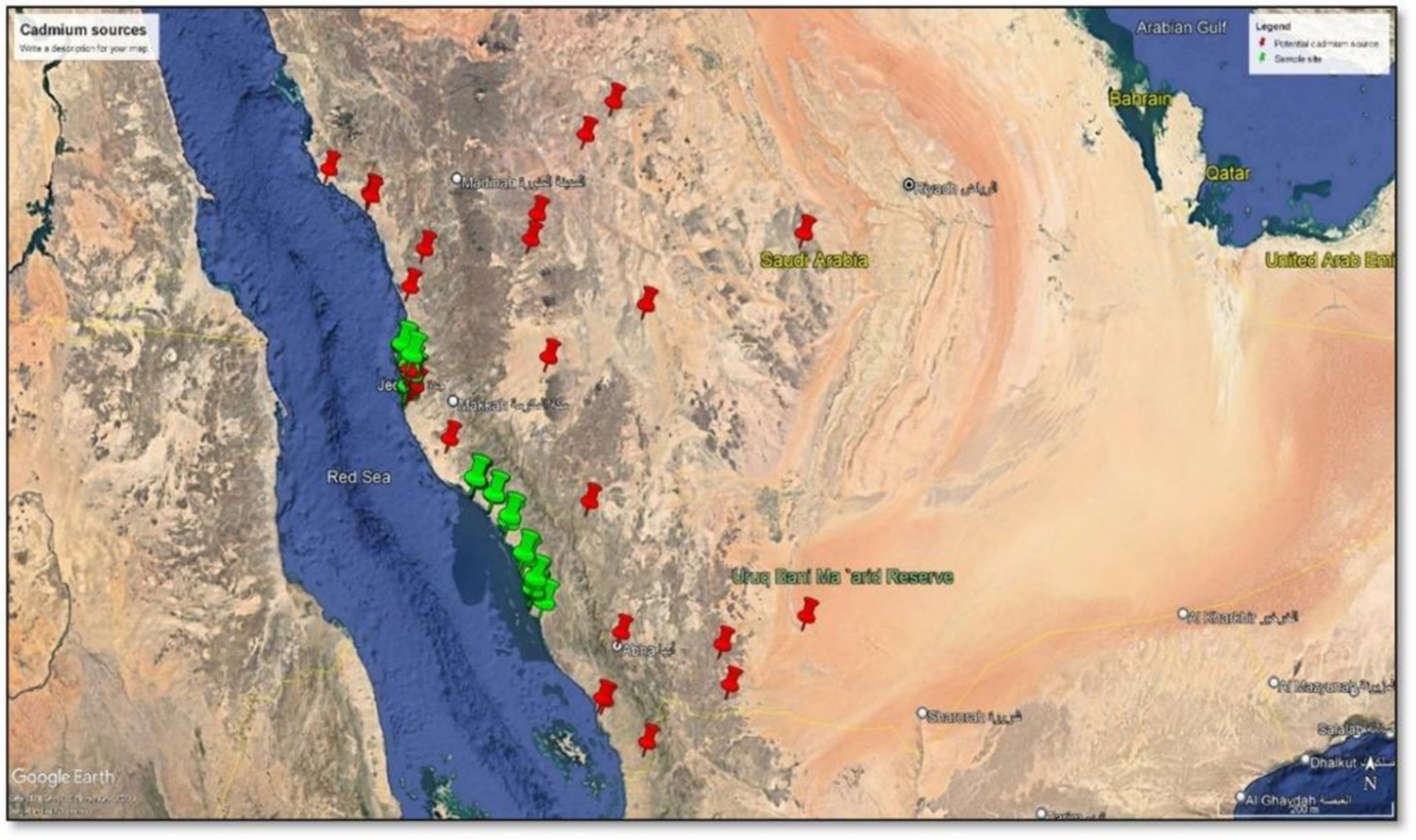
Predicted contamination sources relative to sampling locations. Potential Cd sources are indicated by red marks and sample locations are indicated by green marks.

**Fig 3:**
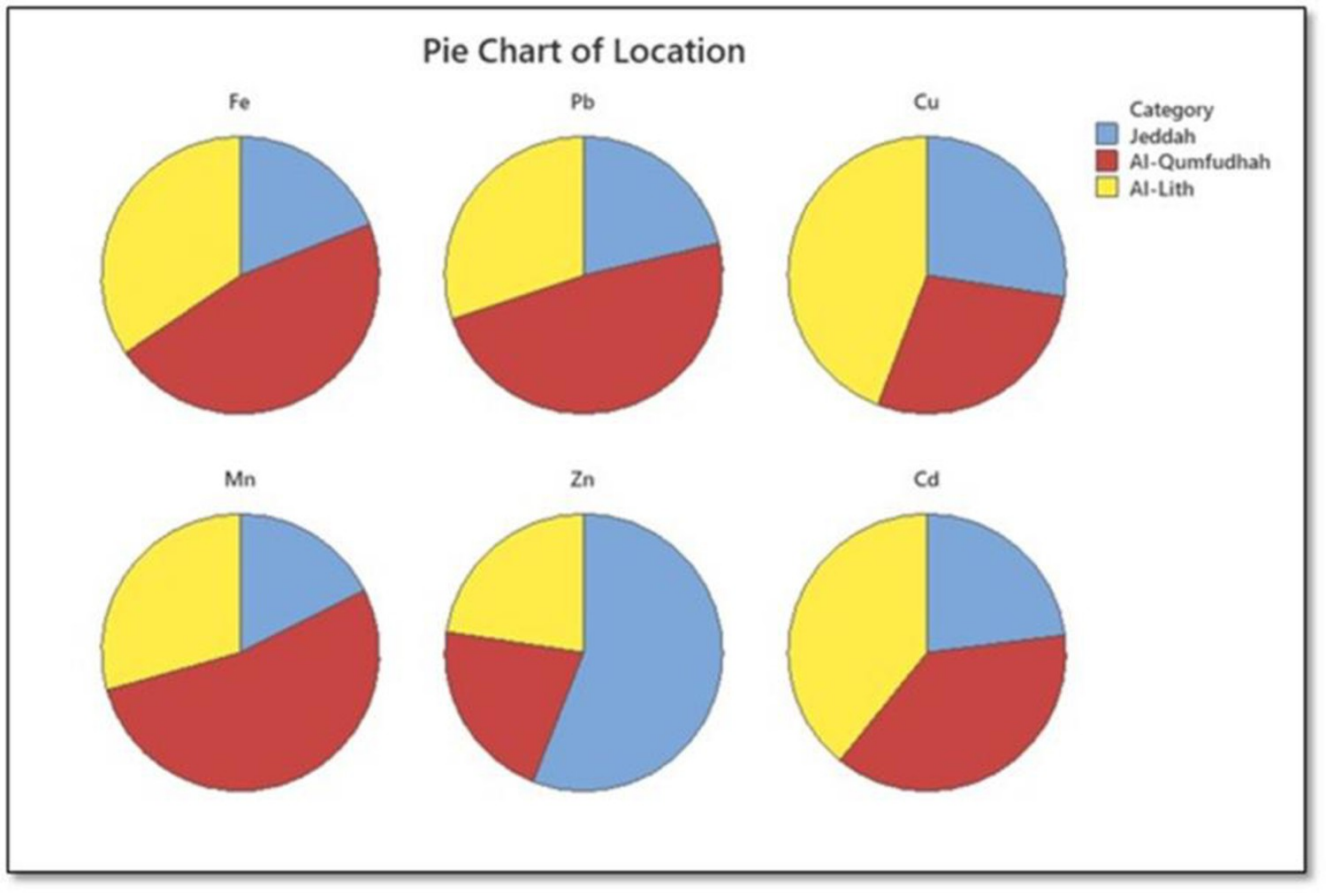
Pie chart showing the relative concentrations of six heavy metals in soil at three different sites. In Al-lith, Cu is the most abundant. In Al-Qunfundah, Pb, Fe, and Mn are abundant. Zn is most abundant in Jeddah. Al-Qunfundah and Al-lith have significant levels of Cd.

**Tables 1−3** also show mean metal concentrations and background levels across various cities. Notably, aside from Fe, Pb, Cu, Mn, and Zn concentrations in beach zones, which were lower than their corresponding background levels, concentrations of Cd surpassed corresponding background levels on all beaches. A high concentration of Cd is probably attributed to natural and anthropogenic sources [18, 26, 27]. Naturally, Cd can be released into the air with subsequent deposition onto beaches through the erosion of rocks [27]. Most rocks around the studied beaches are from the Precambrian basement where sedimentary rocks are exposed (**S1 Fig** in S1 Data) [28]. Notably, these sedimentary rocks possess higher Cd levels (0.01−2.6 mg/kg) [27]. Therefore, the proximity of large rocks near these beaches could be associated with the high levels of Cd found there, together with windblown volcanic emissions. However, natural contributions to the high Cd concentrations are not believed to exceed 30% [29]. Additionally, anthropogenic sources such as deposition of atmospheric particles from burning fossil fuels [26], abrasion from tires and brakes, and emissions from traffic may also contribute to the increased levels of Cd observed in these beach areas, and this can be attributed to extensive development along the western coast and widespread human activities in the area, along with the movement of numerous ships in the Red Sea, which is expected to have a significant impact on the concentrations of heavy metals in general, and Cd specifically [13, 18, 24, 30]. It is noteworthy that near the beaches where samples were taken, there are many cement factories, five big oil refineries, steel mills, three metal refineries, one mine, metals warehouses, water desalination plants, power generation facilities, and various industrial activities (**Fig 2**). Additionally, Fig 3 shows that Al-Qunfundah and Al-lith have significant levels of Cd. The high Cd concentrations are likely the result of a wide range of human activities and multiple sources in the area, from both natural and anthropogenic sources. Human activities have greatly increased cadmium (Cd) levels on Earth, surpassing its natural abundance in the Earth’s crust [31]. Industrial processes, agriculture, and waste disposal have led to human-induced Cd emissions, raising atmospheric Cd levels by two to five times above those from natural sources [31]. If these trends persist, Cd concentrations in soil and water are expected to rise, potentially exceeding safe limits for human consumption and ecosystem health. Cd is known for its long residence time in the environment, with a half-life ranging from 10 to 30 years, contributing to its persistence [32, 33].

Heavy metal levels measured in this study were compared with those recorded for other Saudi cities and various nations (**Table 4**). Al-Qunfudhah has the highest concentration of Fe overall, averaging 13,349 ppm, surpassing Jeddah (5444 ppm) and Al-lith (11,405 ppm) as highlighted in **Tables 1−3**. The variability in Fe levels along the coast stems from the dynamic coastal settings rich in natural sources [34]. For instance, the 2017 Fe measurements for Jeddah beach [18, 35] were lower than our values, highlighting coastal fluctuations. Furthermore, variation in Fe levels can also be attributed to factors such as worn brake pads and vehicle emissions [36]. Elevated Fe levels suggests an increase in vehicular activity releasing Fe through exhaust emissions. Additionally, substantial recent developments in road and building construction in Jeddah, Al-Lith, and Al-Qunfudhah [18] must be considered when attempting to understand the observed trends. Notably, the assigned beaches are situated near major roads, which often experience traffic, particularly on weekends. As a result, Fe may originate from and be transported to the beaches via exhaust emissions and particles from vehicle brake pads [36]. This is likely a primary factor contributing to the elevated Fe levels observed at these beaches, in addition to ongoing construction activities in the vicinity [18].

**Table 4:** Mean heavy metal concentrations (ppm) in coastal Red Sea samples compared with previous studies/countries.

Location	Country	Fe	Pb	Cu	Mn	Zn	Cd	Ref.
Jeddah Beach (this study)	KSA	5444.00	10.87	11.766	84.32	197.3	1.720	_
Al-Lith Beach (this study)	KSA	11405	13.044	21.847	162.30	92.03	3.386	_
Al-Qunfudhah Beach (this study)	KSA	13349	18.15	12.107	253.84	75.61	2.833	_
Jeddah Beach (2017)	KSA	5011.00	24.00	5.70	111.30	54.40	0.11	[18]
Red Sea, Jeddah (2009)	KSA	2351	89.5	20.5	119.4	64.55	3.30	[13]
Red Sea, Rabigh (2009)	KSA	2028	87.21	21.28	153.5	52.40	3.510	[13]
Yanbu, Red Sea (2009)	KSA	2919	79.27	22.07	296.8	74.32	2.90	[13]
Red sea, Jeddah (1996)	KSA	_	_	13.20	23.80	17.95	3.900	[37]
Arabian Gulf (1994)	KSA						1.65	[38]
Gulf of Aden (2006)	Yemen	2454	76.43	59.54	398.5	142.6	_	[39]
Red sea, Yemen (2000)	Yemen	3657	6.91	32.05	42.85	113.3	_	[40]
Al-Hodeidah (2002)	Yemen	_	2.34	6.515	_	21.50	7.40	[41]
Gulf of Aden (2000)	Yemen	_	_	_	_	_	1.25	[42]
Red Sea (2005)	Egypt	9881	_	21.43	235.7	51.4	_	[43]
Abu Kir Bay (1993)	Egypt	_	_	235.4	_	_	_	[44]
El-Mex Bay (2005)	Egypt	_	_	_	_	_	2.85	[45]
Gulf of Aqaba (1987)	Jordan						8.90	[46]
Gulf of Mannar (2003)	India	6500	15.98	_	295.5	72.53	_	[47]
Gulf of Mexico (1999)	Mexico	_	10.21	11.26	230.7	39.82	_	[48]
Florida Bay (2003)	USA	_	_	_	675.5	_	_	[49]
Lavaca Bay (1997)	Hong Kong	_	_	_	_	_	0.23	[50]
Espinho Beach (2006)	Portugal	_	59.6	_	371.4	96.6	_	[14]

However, along coastal regions of Jeddah, Al-lith, and Al-qunfudhah, the average Pb content was moderate (10.873, 13.033, and 18.195 ppm, respectively). These observed lead concentrations were comparatively lower than those reported in other reference locations and international cities such as the Gulf of Mexico (Mexico), the Gulf of Mannar (India), Yanbu, and Rabigh (Saudi Arabia; KSA). The ban on leaded gasoline in these areas (Jeddah, Al-Lith, and Al-Qunfudhah) may account for the reduced Pb quantities, potentially signaling an enhancement in beach soil quality [3, 14, 18]. The average Cu concentration in Jeddah (11.76 ppm) and Al-Qunfudhah (12.107 ppm) was lower than in Al-Lith (21.847 ppm) and similar between Jeddah and Al-Qunfudhah. This indicates potential anthropogenic influences, such as brake housing dust and crushed brake abrasion materials in urban environments, contributing to Cu levels in Al-Lith City [24].

The mean Mn concentration in Al-Qunfudhah (253.84 ppm) surpassed levels found in Jeddah and Al-Lith (84.32 and 162.30 ppm, respectively) but was lower than cities worldwide including Florida Bay, USA (675.5 ppm) and beach sediments in Espinho, Portugal (371.4 ppm). Variations in motor vehicles and industrial activities likely contributed to these differences, although demographic differences across these cities might also have an impact [51, 52]. Interestingly, the Zn mean concentration on Jeddah beach (197.38 ppm) was markedly higher than in other cities within this study, and in significant areas including the Red Sea (Egypt) and Al-Hodeidah (Yemen)as indicated in **Table 4**. On the other hand, the Zn concentration in Jeddah was higher than in beach sediments from Espinho (Portugal) and Gulf of Suez beach (Egypt). Multiple studies have associated increased traffic and tire wear with higher Zn concentrations [53]. Throughout this investigation, moderate to low average amounts of Fe, Pb, Cu, Mn, and Zn were observed, highlighting the general range of these metals in the studied areas.

### 3.2. Correlation coefficient analysis

**Fig 4:**
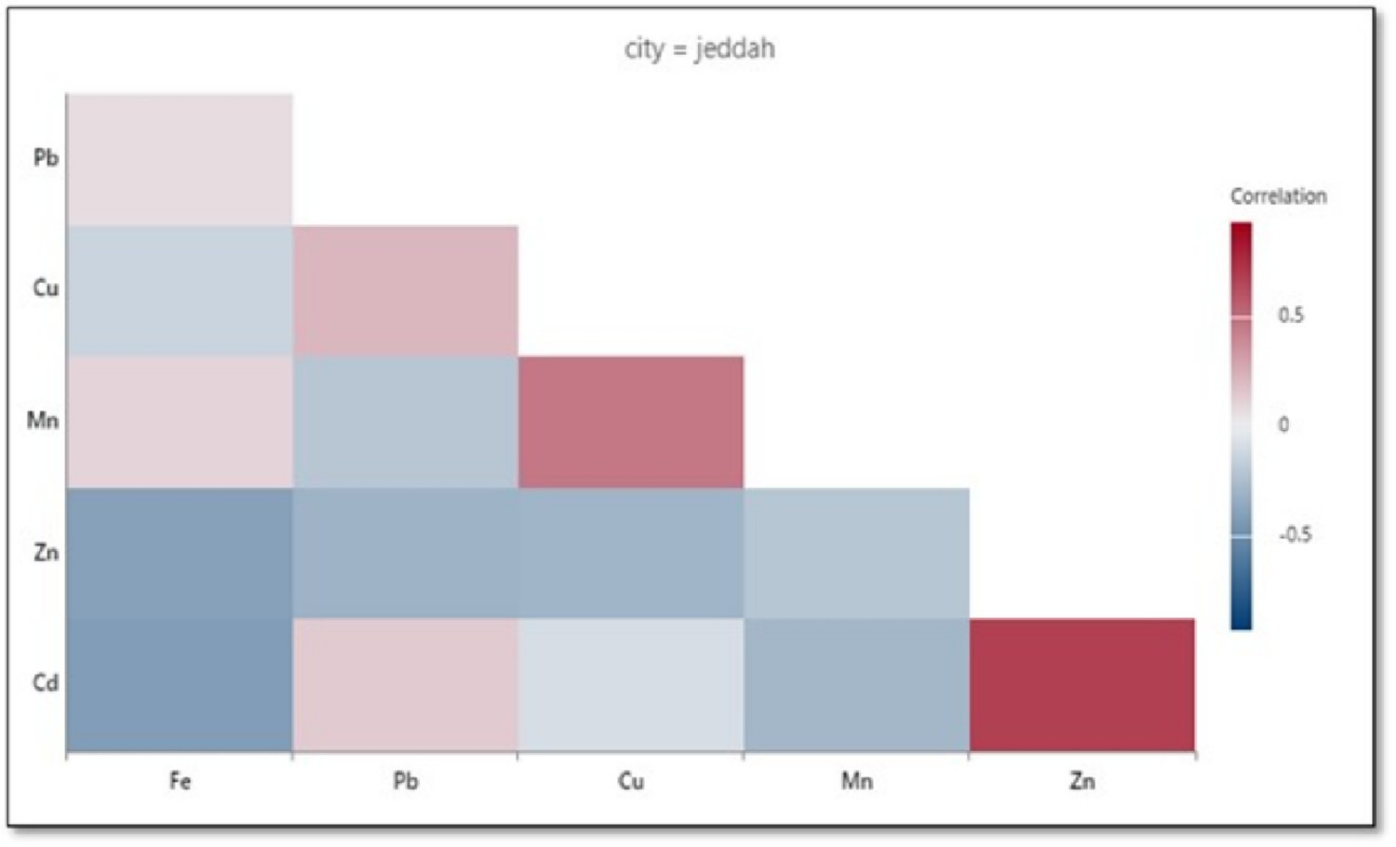
Correlogram of Fe, Pb, Cu, Mn, Zn, and Cd at Jeddah beach.

**Fig 5:**
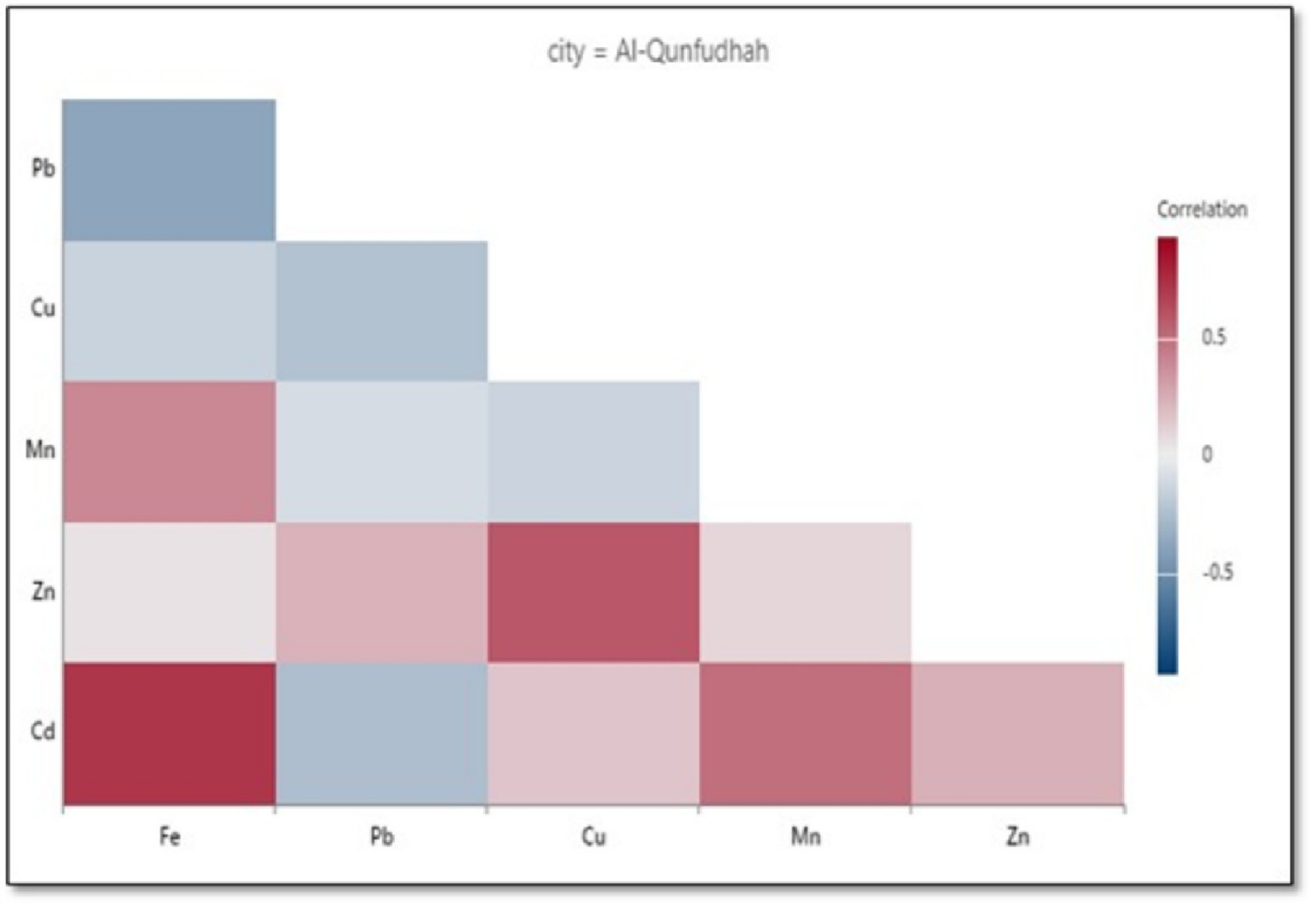
Correlogram of Fe, Pb, Cu, Mn, Zn, and Cd at Al-Qunfudah beach.

**Fig 6:**
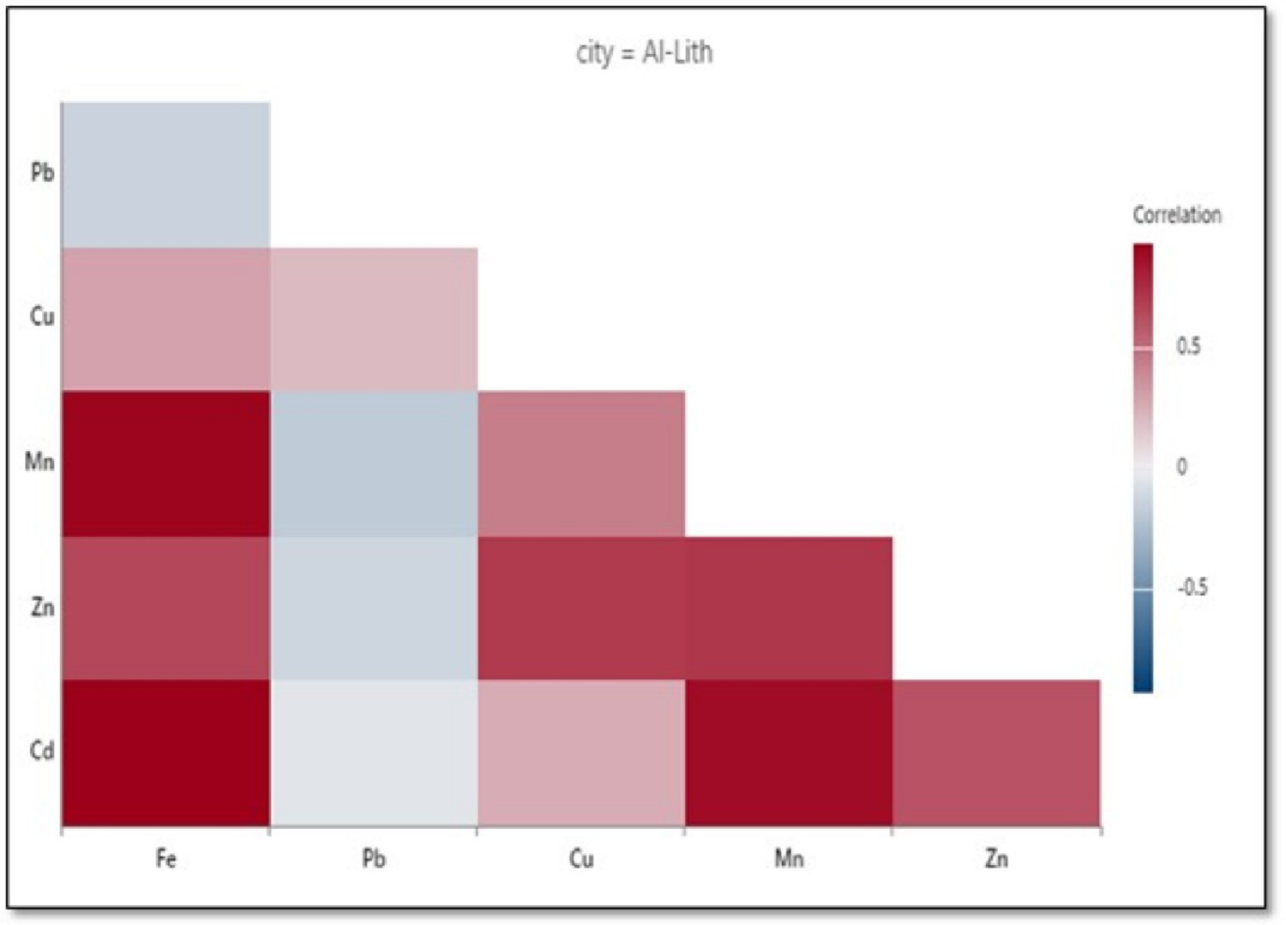
Correlogram of Fe, Pb, Cu, Mn, Zn, and Cd at Al-Lith beach.

Individual correlation matrix coefficients depicted in **Figs 4−6** for each city reveal relationships among heavy metals, with both positive and negative correlations. For example, as shown in **Fig 4** for Jeddah, a strong positive correlation was evident for Zn-Cd but there was almost no correlation for Cd-Fe. On the other hand, a strong positive correlation was found for Cd-Fe in Al-Qunfudah (**Fig 5**) and Al-Lith (**Fig 6**). A strong positive correlation indicates that metals were emitted either from shared anthropogenic sources such as vehicular emissions, traffic activities, and industrial processes, or from different sources at similar rates [21].

Accumulative correlation matrix coefficients between selected heavy metals for all beaches are listed in **Table 5**. The results reveal positive and negative correlations between metals. Strong positive correlations were observed for Fe-Pb, Fe-Mn, Fe-Cd, Pb-Mn, and Cu-Cd. As mentioned previously, positive correlations suggest either emissions from shared anthropogenic sources, or different sources with the same emission rates [21]. Moderate positive correlations were identified for Cd-Pb and Cd-Mn, indicating common origins from manufacturing activities and motor vehicles [54, 55]. On the other hand, a weak correlation was found for Fe-Cu, suggesting distinct anthropogenic origins or sources for these metals. Interestingly, a negative correlation or no correlation was observed between Zn and all other elements, as well as for Cu-Pb and Cu-Mn. These results suggests that these elements are obviously emitted from different sources. Moreover, Cd coexists with other heavy metals including Pb, Cu, and Zn as a natural component of rocks and sediments, and this coexistence is affected by many factors including soil pH, organic matter, and the type of human activities in the area. The main activities resulting in high accumulation of Cd and other heavy metals are mining and smelting [56].

**Table 5:** Pearson’s accumulative correlation matrix between heavy metals for all beaches.

Variables	Metals					
	Fe	Pb	Cu	Mn	Zn	Cd
Fe	1					
Pb	**0.870861**	1				
Cu	0.309975	-0.19737	1			
Mn	**0.945546**	**0.983426**	-0.0163	1		
Zn	-0.99356	-0.80954	-0.4157	-0.902	1	
Cd	**0.841804**	0.467776	**0.77413**	0.620	-0.897	1

### 3.3. Principal component analysis (PCA)

The results of correlation analysis did not allow us to definitively determine the origins of metals. Therefore, to further explore where the various heavy metals identified in this study came from, PCA was conducted. In PCA, a small number of latent factors, referred to as PCs, are extracted to assess relationships between observed variables. PCA is a commonly employed technique for data reduction [57]. Kaiser normalization determines the number of components to retain, and components with eigenvalues >1 were chosen. A component’s contribution is considered substantial when the eigenvalue associated with it is >1. In our PCA, the six heavy metals originating from Jeddah, Al-Qunfundah, and AL-lith served as variables. Two PCs with eigenvalues greater than unity were generated by the analysis, accounting for a cumulative variance of 60.8% (**Table 6**). The initial component (PC1) accounted for the greatest proportion of variance (40.3%), with PC2 accounting for 20.5% of the variance. **Table 7** displays the factor loadings, which serve as an indicator of the variables’ significance. For this analysis, loading values >0.50 were deemed acceptable.

**Table 6:** Eigen analysis of the PCA matrix.

Eigenvalue	2.4179	1.2275
Proportion	0.403	0.205
Cumulative	0.403	0.608

**Table 7:** Loading factors.

Eigenvectors	First component	Second component
Variable	PC1	PC2
Fe	0.558	0.072
Pb	0.035	0.549
Cu	0.278	-0.243
Mn	0.563	0.091
Zn	-0.203	-0.7
Cd	0.501	-0.369

**Fig 7:**
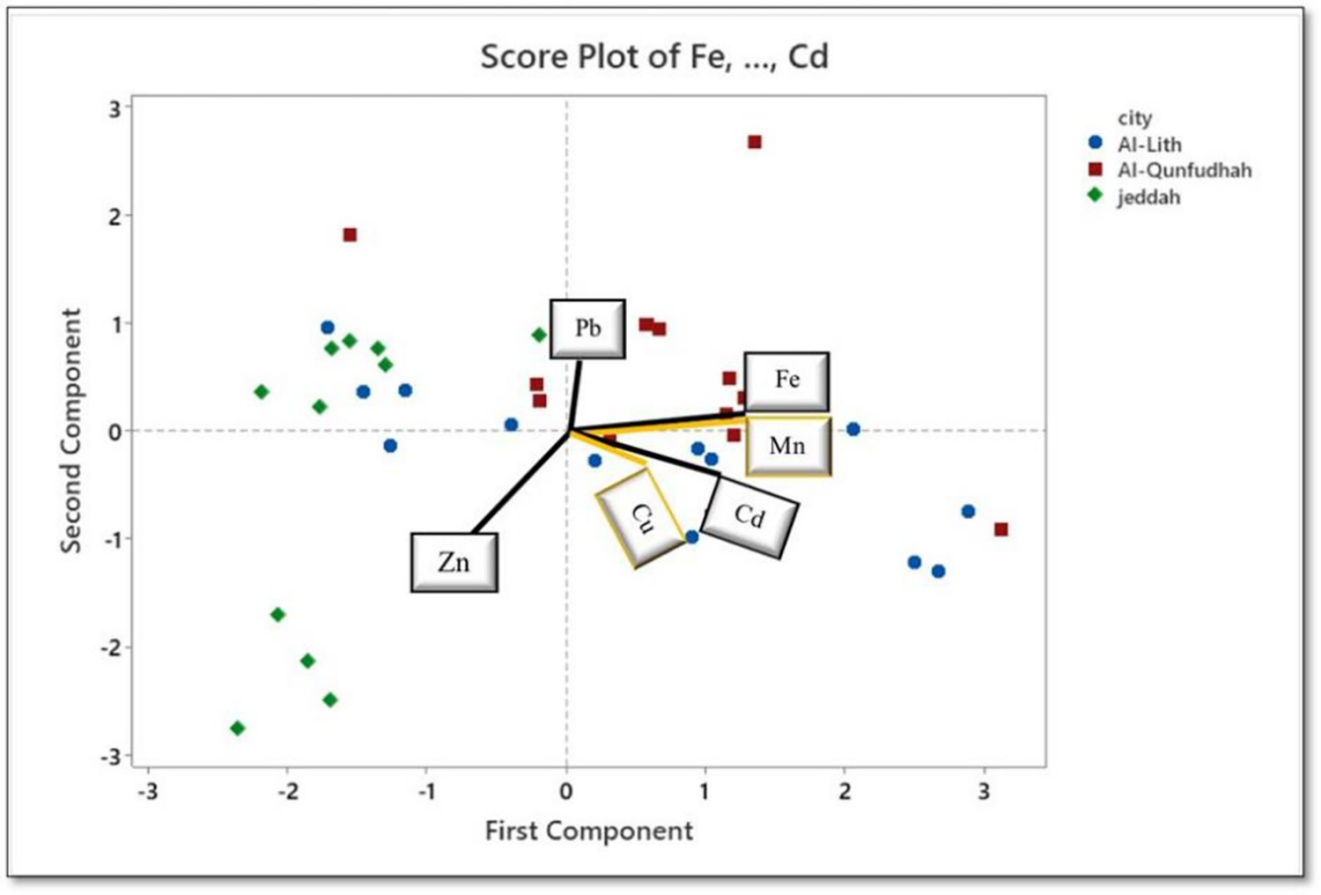
Biplot of PCA factors for all selected metals on all beaches tested.

**Fig 7** Biplot of PCA factors, PC1 and PC2, for all heavy metals on all beaches tested. The highest levels of Pb, Fe, and Mn were accumulated on Al-Qundudah beach, while Cd and Cu were accumulated mainly on Al-Lith beach, and Zn was accumulated only on Jeddah beach. These results were confirmed using the pollution index (PI) factor in the next section.

Positive loadings were observed for Mn, Fe, and Cd in PC1 (**Fig 7**). As described above, soil samples from Jeddah and AL-lith demonstrated a notable inverse correlation with Zn and Pb (**Figs 4−6**). PC2 included Zn and Pb, with Zn and Pb having negative and positive loading, respectively. Fe, Mn, and Cd were significantly correlated with soil samples from AL-lith (**Fig 6**).

### 3.4. Cluster analysis

**Fig 8:**
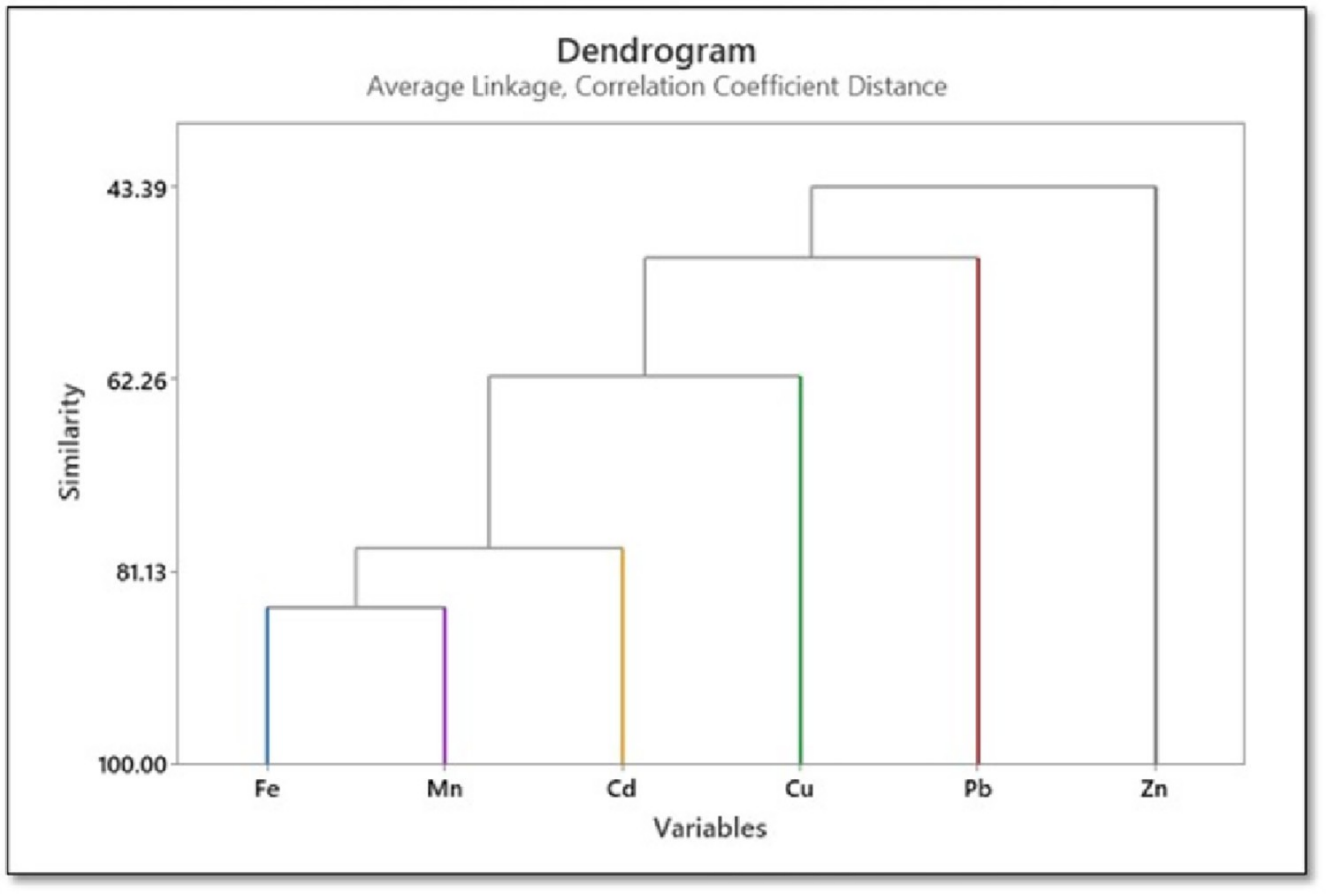
Dendrogram of Fe, Pb, Cu, Mn, Zn, and Cd.

In order to validate the results of PCA, cluster analysis was performed, and the results are displayed in a dendrogram in **Fig 8**. This representation of data in two dimensions using color to indicate values facilitates rapid visual understanding. The dendrogram was generated with a final clustering scheme of six nodes, and different colors indicating each final cluster. The dendrogram was cut at a similarity level of 43.39%. There would be fewer final clusters and a lower degree of similarity if the dendrogram were trimmed higher, and more final clusters but a higher similarity level if the dendrogram was trimmed lower. Hierarchical clustering/cluster analysis results are arranged in rows and columns. Cluster analysis makes it easier to classify cases (objects) by highlighting similarities within a class and differences between classes. Cluster analysis helps interpret data and identify patterns [58]. The results comprise six variables; a new cluster was created in step 1 by joining two existing clusters, represented in the worksheet as variables 1 and 4. With a similarity level of 84.6787% and a distance level of 0.30643 (**Table 8**), this partitioned the data into five clusters. In the final step, all variables were joined into a single cluster. Fe, Mn, and Cd for a tight cluster (**Fig 8**). Heavy metal clustering supported the PCA results. Six main categories were used to classify the heavy metals. In the second group, visible in the loading plot, Fe and Mn formed a small group (**Fig 8**).

**Table 8:** Amalgamation steps and final partitioning in cluster analysis.

Step	Number of clusters	Similarity level	Distance level	Clusters joined		New clusters	Number of obs. in new clusters	Variables	
1	5	84.6787	0.30643	1	4	1	2	**Cluster 1**	Fe
2	4	78.8678	0.42264	1	6	1	3	**Cluster 2**	Pb
3	3	62.0149	0.75970	1	3	1	4	**Cluster 3**	Cu
4	2	50.3430	0.99314	1	2	1	5	**Cluster 4**	Mn
5	1	43.3935	1.13213	1	5	1	6	**Cluster 5**	Zn

### 3.5. Pollution assessment

#### 3.5.1. Enrichment factor (EF)

EF analysis serves as a valuable tool for assessing the proportions of elements within analyzed samples. It involves comparing the observed elemental composition to that of the Earth’s crust, with elements such as Al, Sc, Ba, and Fe commonly used as reference elements. Given that soil is considered a primary source of Fe in aerosols, Fe levels present in the Earth’s crust were utilized as a reference in this study to compute EF as shows in Eq 1 [56, 57]:

$$\mathrm{EF}=\frac{(C{\mathrm{Me}/\mathrm{CFe})}_{\mathrm{Sample}}}{{\left(C\mathrm{Me}/\mathrm{CFe}\right)}_{\mathrm{Crust}}}$$ (1)

where (CMe)_sample_ represents the element’s concentration in the sample of beach soil, (CMe)_crust_ represents the element’s concentration in the crust, and (CFe) represents the element’s concentration in both the sample and the crust. EF analysis provides a scale to assess the degree of contamination or deviation from the natural composition of the Earth’s crust for specific elements within the studied samples. The five contamination levels based on EF values for heavy metals are as follows [59, 60]:

1. EF < 2 denotes mineral deficiency.
2. EF = 2–5 denotes moderate enrichment.
3. EF = 5–20 denotes significant enrichment.
4. EF = 20–40 denotes very high enrichment.
5. EF > 40 denotes extremely high enrichment.

**Fig 9:**
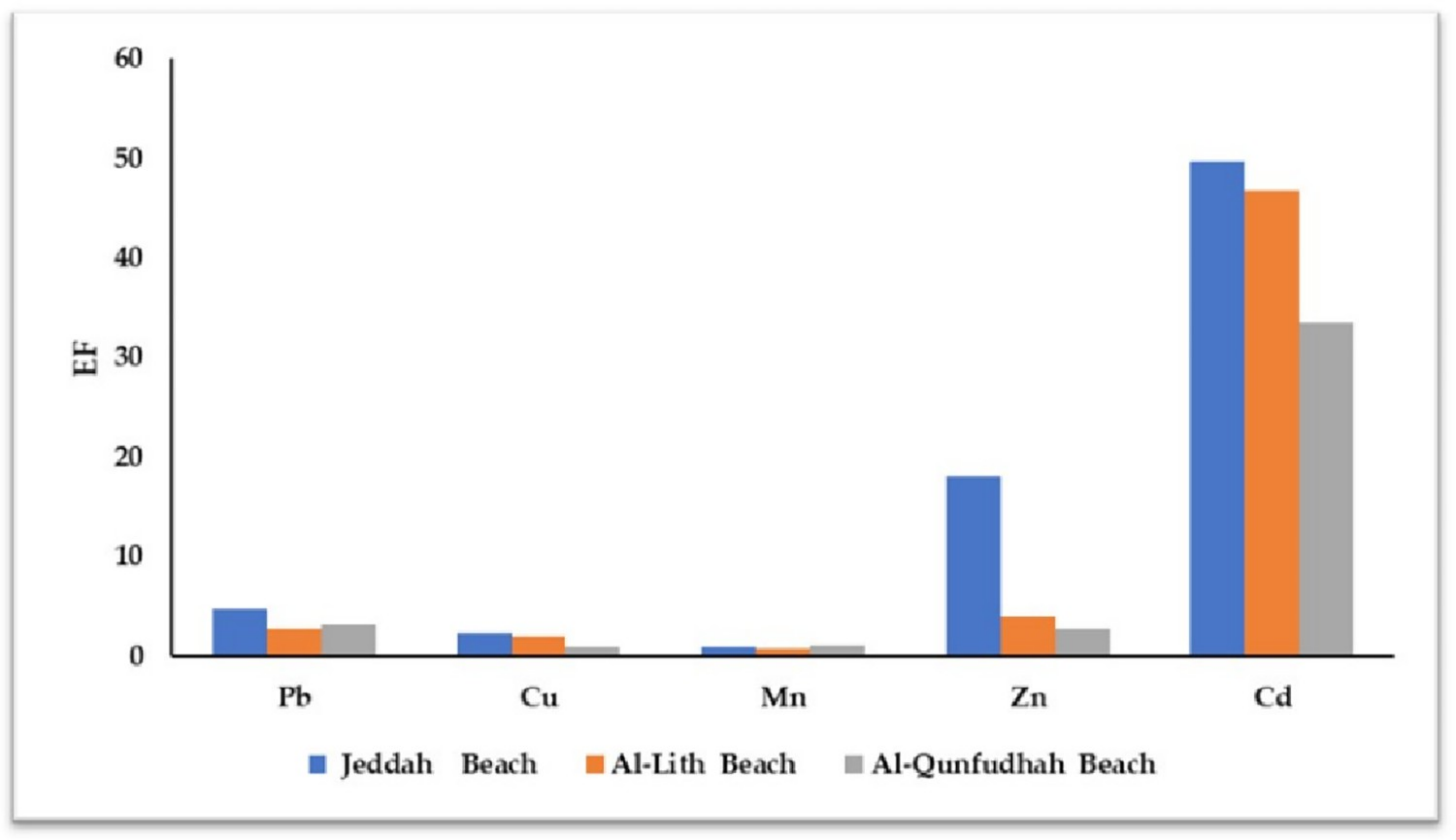
EF value analysis of heavy metal concentrations in beach soils.

**Table 9** and **Fig 9** show EFs corresponding to the selected heavy metals across different beaches. This visual representation and tabulated data showcase how each EF values for each heavy metal fall into these contamination categories, providing a clear understanding of the level of enrichment or depletion observed in samples compared to the natural composition of the Earth’s crust.

**Table 9:** Crustal average EF for all beach soil samples.

Location	Pb	Cu	Mn	Zn	Cd
Jeddah	4.71	2.26	0.86	18.0	49.7
Al-Lith	2.69	2.01	0.79	4.01	46.7
Al-Qunfudhah	3.21	0.95	1.05	2.81	33.4

EF analysis revealed that most of the metals studied exhibited enrichment across all coastal areas (except Mn), predominantly due to anthropogenic factors. Average EF values for heavy metals were ordered Cd > Zn > Pb > Cu > Mn (high to low). Jeddah beach displayed the highest significant average EF values for Cd, Zn, Pb, and Cu (49.7, 18.0, 4.71, and 2.26, respectively). The markedly elevated EF values for Cd, Zn, Pb, and Cu suggest that these metals originate from sources other than the earth’s crust. These results strongly suggest a common origin for these substances, likely stemming from vehicular, traffic, and industrial activities. Conversely, Mn displayed consistently lower EF values across the board, indicating that anthropogenic influences on this element might not be as pronounced as they are for the other metals studied. This disparity in EF values highlights the differing degrees of anthropogenic impact on the different heavy metals studied.

#### 3.5.2. Pollution index (PI)

PI is a measure obtained by dividing the concentration of each metal by its background concentration value according to the following formula [60]:

$$\mathrm{PI}=\frac{C_{\mathrm{metal}}}{C_{\mathrm{background}\;\mathrm{value}}}$$ (2)

where C_Metal_ represents the metal concentration measured on the beach, and C _background value_ indicates the background concentration value of the metal. PI is categorized into three groups: low (PI ≤1), moderate (1 < PI ≤ 3), and high (PI > 3) [61, 62].

**Table 10** lists the PI values for six heavy metals assessed across different beaches. For most metals, all three beaches displayed PI values <1, except for Zn on Jeddah beach (as shown in PCA) and Cd and on all beaches, which surpassed this threshold. Notably, Cd showed a consistently high PI across all beaches, likely attributed to natural and anthropogenic sources [63], as described above in section 3.1. This finding is in good agreement with the results in **Tables 1−3** where the concentrations of Cd on all beaches surpassed the corresponding background levels. Additionally, the elevated PI for Zn in Jeddah is in good agreement with the PCA results and our previous study on Jeddah City [18], where Zn exhibited the highest PI value due to its prevalence in tire wear, linked to the substantial traffic in this urban area.

**Table 10:** Pollution index (PI) and integrated PI (IPI) for all beach soil samples.

	Location	PI						IPI
		Fe	Pb	Cu	Mn	Zn	Cd	
1	Jeddah	0.12	0.54	0.26	0.01	2.08	5.74	1.45
2	Al-Lith	0.24	0.65	0.48	0.19	0.96	11.3	2.30
3	Al-Qunfudhah	0.28	0.91	0.26	0.29	0.79	9.44	1.99

#### 3.5.3. Integrated PI (IPI)

IPI, determined using a modified version of the generalized equation, is the overall level of contamination [54]. IPI is the total IP for a particular set of pollutants divided by the total number of pollutants assessed, according to the following equation:

$$IPI=\frac{\sum_{i=1}^{i=n}IP}{n}$$ (3)

where i is the i^th^ element and n is the number of studied elements. IPI is divided into four related categories: low (IPI ≤ 1), moderate (1< IPI ≤ 2), high (2 < IPI ≤ 5), and extremely high (IPI > 5). [58, 64].

IPI for heavy metals was 2.30, 1.99, and 1.45 for Al-Lith, Al-Qunfudhah, and Jeddah, respectively (**Table 10**). The IPI values for beaches indicated a high level of heavy metal contamination in Al-lith beach, which could be attributed to more anthropogenic sources than for other locations [65]. On the other hand, the IPI values for heavy metals across all beaches were 0.21, 0.70, 0.33, 0.16, 1.28, and 8.83 for Fe, Pb, Cu, Mn, Zn, and Cd, respectively. The four IPI categories indicate that Cd stands out as an extremely highly prevalent contaminant across all beaches. This prominence of Cd could be traced back to anthropogenic sources influencing its presence. Collectively, these findings highlight the widespread nature of Cd pollution in all beach locations, raising concerns about potential serious public health issues such as chronic kidney disease (CKD) [66], osteomalacia, and osteoporosis [67].

### 3.6. Potential human health risks of selected metals in beach samples

To assess potential chronic (non-carcinogenic) impacts resulting from ingesting seashore materials, this study determined the average daily dose (ADD) of heavy metals and their associated risks. ADD values (μg/g day^-1^) for specific metals on beaches were calculated using the following equation [68]:

$$\mathrm{ADD}=\frac{\left(C\;x\;C_{F}\;x\;\mathrm{IR}\;x\;E_{F}\;x\;E_{D}\right)}{\left(\mathrm{BWx}\;\mathrm{AT}\right)}$$ (4)

where ’C’ represents the concentration of heavy metals (μg/g), IR corresponds to the dust ingestion rate, and the recommended ingestion rate for dust is set at 60 mg.day^-1^ for children and 30 mg.day^-1^ for adults [69]. Estimated average body weight (BW) is considered 15 kg for children and 70 kg for adults. Exposure frequency (E_F_) is assumed to be 350 days per year. In this study, exposure duration (E_D_) is considered 6 years for children and 24 years for adults [70–73]. For non-carcinogens, the average time (AT) equals E_D_ multiplied by 365 days, and C_F_ represents the conversion factor, which is 10^−6^ kg.mg^-1^.

Furthermore, the potential chronic effects of each metal were assessed by comparing the level of exposure (oral dust ingestion) over a specified period with a reference dose (RfD) established for a similar exposure duration]. The term hazard quotient (HQ) denotes this ratio. Hazard index (HI) was computed to assess the overall risk of chronic damage posed by multiple chemicals [70, 74]. HQ and HI were calculated using the following formula:

$$HQi=\frac{ADD}{RfDi}$$ (5)

HI=∑HQi (6)

where RfDi is obtained from screening levels [71, 75]. HI serves as an indicator of the probability of chronic effects; high HI suggests an increased probability of experiencing chronic effects due to exposure to chemicals.

The study evaluated the human health risks posed by metal exposure on Jeddah beach using HI and HQ. Tables 11–13 display parameter values alongside outcomes for HQ, HI, and ADD for all metals [76]. The potential health risks associated with ingesting beach debris orally was considered for both children and adults. HQ values for children and adults were ordered Fe > Pb > Zn > Mn > Cd > Cu at Jeddah beach. Similarly, at Al-Lith and Al-Qunfudhah beaches, HQ values were ordered Fe > Pb > Mn > Cd > Cu > Zn for children and adults. HI values for Jeddah, Al-Lith, and Al-Qunfudhah beach samples were determined as 0.047, 0.084, and 0.102 for children, and 0.005, 0.010, and 0.011 for adults, respectively. Notably, HQ and HI values were all <1. This suggests that orally ingesting these heavy metals from the beaches poses no significant risk of long-term side effects for both children and adults.

**Table 11:** Health risks from heavy metals on Jeddah beach.

Metal	Mean C (μg/g)	RfD_j_ (μg/g day^-1^)	ADD (μg/g day^-1^)		HQ		HI	
			Children	Adult	Children	Adult	Children	Adult
Fe	5444	7.0 x 10^−1^	2.18x 10^−2^	2.23x 10^−3^	3.11x 10^−2^	3.19x 10^−3^		
Pb	10.87	3.5 x10^-3^	4.17x 10^−5^	4.46x 10^−6^	1.19x 10^−2^	1.27x 10^−3^		
Cu	11.76	4.0 x 10^−2^	4.51x 10^−5^	4.83x 10^−6^	1.12x 10^−3^	1.20x 10^−4^		
Mn	84.32	1.4 x 10^−1^	3.23x 10^−4^	3.46x 10^−5^	2.31x 10^−3^	2.47x 10^−4^		
Zn	197.3	3.0 x 10^−1^	7.57x 10^−4^	8.11x 10^−5^	2.52x 10^−3^	2.70x 10^−4^		
Cd	1.720	3.0 x 10^−3^	6.59x 10^−6^	7.07 x 10^−7^	2.19 x 10^−3^	2.36 x 10^−4^	**0.047**	**0.005**

**Table 12:** Health risks from heavy metals on Al-Lith beach.

Metal	Mean C (μg/g)	RfD_j_ (μg/g day^-1^)	ADD (μg/g day^-1^)		HQ		HI	
			Children	Adult	Children	Adult	Children	Adult
Fe	1140	7.0 x 10^−1^	4.37x 10^−2^	4.68x 10^−3^	6.24x 10^−2^	6.69x 10^−3^		
Pb	13.04	3.5 x10^-3^	5.00x 10^−5^	5.36x 10^−6^	1.42x 10^−2^	1.53x 10^−3^		
Cu	21.84	4.0 x 10^−2^	8.37x 10^−5^	8.97x 10^−6^	2.09x 10^−3^	2.24x 10^−4^		
Mn	162.3	1.4 x 10^−1^	6.22x 10^−4^	6.66x 10^−5^	4.44x 10^−3^	4.76x 10^−4^		
Zn	92.03	3.0 x 10^−1^	3.52x 10^−4^	3.78x 10^−5^	1.17x 10^−3^	1.26x 10^−4^		
Cd	3.386	3.0 x 10^−3^	1.29x 10^−5^	1.39 x 10^−6^	4.32 x 10^−3^	4.63 x 10^−4^	**0.084**	**0.010**

**Table 13:** Health risks from heavy metals on Al-Qunfudhah beach.

Metal	Mean C (μg/g)	RfD_j_ (μg/g day^-1^)	ADD (μg/g day^-1^)		HQ		HI	
			Children	Adult	Children	Adult	Children	Adult
Fe	13349	7.0 x 10^−1^	5.12x 10^−2^	5.48x 10^−3^	7.31x 10^−2^	7.83x 10^−3^		
Pb	18.195	3.5 x10^-3^	6.97x 10^−5^	7.47x 10^−6^	1.99x 10^−2^	2.13x 10^−3^		
Cu	12.107	4.0 x 10^−2^	4.64x 10^−5^	4.97x 10^−6^	1.16x 10^−3^	1.24x 10^−4^		
Mn	253.84	1.4 x 10^−1^	9.73x 10^−4^	1.04x 10^−4^	6.95x 10^−3^	7.43x 10^−4^		
Zn	75.614	3.0 x 10^−1^	2.90x 10^−4^	3.10x 10^−5^	9.66x 10^−4^	1.03x 10^−4^		
Cd	2.8333	3.0 x 10^−3^	1.08x 10^−5^	1.16 x 10^−6^	3.62 x 10^−3^	3.87 x 10^−4^	**0.102**	**0.011**

It is crucial to acknowledge that over time, the bodies of mammals can accumulate harmful heavy metals, potentially impacting their health. Therefore, the potential health risks associated with exposure to heavy metals on beaches, both for adults and children, should not be disregarded. However, based on this study, heavy metals on all beaches are not considered to have an adverse impact on the health of children or adults.

## 4. Conclusion

Studies on heavy metal concentrations aimed to assess the presence of pollutants and associated health risks for three distinct coastal cities—Jeddah, Al-Lith, and Al-Qunfudhah—located along Saudi Arabia’s Red Sea coast. Interestingly, unlike Fe, Pb, Cu, Mn, and Zn concentrations in the beach zones, which were below their respective background levels, Cd concentrations exceeded corresponding background levels on all beaches. These elevated Cd levels are likely attributed to anthropogenic sources. EF analysis was employed to compare natural and human-induced sources of heavy metal presence relative to the abundance in the Earth’s crust. Average EF values were ordered Cd > Zn > Pb > Cu > Mn. Notably, all metals examined, except for Mn, showed enrichment across all locations, indicating sources beyond the Earth’s crust for these metals. PCA was conducted for all heavy metals on all beaches. The results indicated that the highest levels of Pb, Fe, and Mn were accumulated on Al-Qundudah beach, while Cd and Cu were mainly accumulated on Al-Lith beach, and Zn was accumulated on Jeddah beach. Pb, Fe, and Mn were grouped on Al-Qundudah beach, while Cd and Cu were grouped on Al-Qundudah and Al-Lith beaches, and Zn was grouped only on Jeddah beach. Furthermore, a thorough investigation into pollution and health risks across these beaches was carried out. IPI and PI were used for contamination assessment, and PI values highlighted extremely high levels of Cd pollution on all three beaches. The overall pollution assessment revealed elevated levels of some heavy metals, particularly Cd, across all beaches, necessitating immediate attention due to potential health risks for both adults and children. This study serves as a foundation for further exploration of potential health issues in these beach areas such as chronic kidney disease (CKD) [65, 68], osteomalacia, and osteoporosis [66, 77].

## Supporting information

S1 Data Supplementary data includes all raw data. (DOCX)
