# Supplementary material for: Pollution and health risk assessments related to heavy metals on three prominent beaches in Makkah Province, Kingdom of Saudi Arabia: Concerning levels of cadmium pollution
Source: PLoS One. 2024 Oct 21;19(10):e0311189. doi: 10.1371/journal.pone.0311189 (PMC11493290; doi:10.1371/journal.pone.0311189)
Supplement: S1 Data — (DOCX) [file pone.0311189.s001.docx]

**“Supplementary Data”**

Pollution and health risk assessments related to heavy metals on three prominent beaches in Makkah Province, Kingdom of Saudi Arabia: Concerning levels of cadmium pollution

**Abdulaziz Alayyafi ^3^, Mohammad Ebqa’ai ^1,2*^, Omar Alboqai ^4^, Ahmed Abotaleb ^5^, Ahmed Eldesoky ^3,6^ and Abeer El Shahawy ^5*^**

^1^ Department of Chemistry, Physics, and Engineering, Cameron University, Lawton, Oklahoma 73505, USA.

^2^ Department of Chemistry, Oklahoma State University, Stillwater, Oklahoma 74078, USA.

^3^ Department of Chemistry, University College in Al-Qunfudah, Umm Al-Qura University, 21912, Saudi Arabia.

^4^ Department of Nutrition and food Science, Faculty of Agriculture, Jerash University, Jerash, Jordan.

^5^ Department of Civil Engineering, Faculty of Engineering, Suez Canal University, Ismailia 41522, Egypt.

^6^ Chemical Engineering Department, High Institute of Engineering &Technology, New Damietta 34517, Egypt.

**Table of content**

| Contents | Page  No. |
| --- | --- |
| Table S1: Raw data of Figure 1: Exact locations for each sample collected using Google earth | S2 |
| Fig S1. A spatial map showing the geology of the Arabian Peninsula | S3 |
| Table S2: Raw data of heavy metal concentrations (ppm) on Jeddah beach | S4 |
| Table S3: Raw data of heavy metal concentrations (ppm) on Al-Qunfudhah beach | S4 |
| Table S4: Raw data of heavy metal concentrations (ppm) on Al-Lith beach | S5 |

To draw the maps of samples position and the expected pollution source, the accurate location of each sample was specified using easting and northing values and they were defined in google earth pro software as points. The location of each pollution source is marked using the search feature in the software where the name of each potential pollution source was the input.

| **Samples** | **Jeddah** | | **Al-Lith** | | **Al-Qunfudhah** | |
| --- | --- | --- | --- | --- | --- | --- |
|  | **Easting** | **Northing** | **Easting** | **Northing** | **Easting** | **Northing** |
| S1 | 39°08'01.9" | 21°15'14.5" | 40°14'38.6" | 20°09'17.7" | 41°14'22.1" | 18°40'23.7" |
| S2 | 39°06'45.9" | 21°17'27.4" | 40°15'02.5" | 20°09'07.0" | 41°23'26.0" | 18°32'04.5" |
| S3 | 39°06'16.3" | 21°18'47.3" | 40°15'12.5" | 20°08'43.9" | 41°13'43.8" | 18°47'31.0" |
| S4 | 39°06'35.5" | 21°19'45.7" | 40°15'34.0" | 20°08'26.3" | 41°14'21.3" | 18°51'02.7" |
| S5 | 39°07'07.3" | 21°19'56.6" | 40°15'42.4" | 20°08'16.0" | 41°14'11.3" | 18°48'52.2" |
| S6 | 39°06'21.2" | 21°37'06.1" | 40°15'47.5" | 20°08'01.0" | 41°14'50.9" | 18°39'04.8" |
| S7 | 39°06'23.8" | 21°36'57.3" | 40°16'19.2" | 20°07'07.8" | 41°14'05.4" | 18°40'48.1" |
| S8 | 39°08'21.2" | 21°45'54.7" | 40°16'30.1" | 20°06'56.7" | 41°13'07.5" | 18°41'45.6" |
| S9 | 38°58'44.5" | 21°53'44.4" | 40°32'44.0" | 19°57'18.7" | 41°05'06.3" | 19°06'54.1" |
| S10 | 38°58'36.5" | 21°53'42.7" | 40°32'53.2" | 19°56'57.8" | 41°05'09.4" | 19°06'35.8" |
| S11 | 38°58'14.9" | 21°52'58.7" | 40°32'57.6" | 19°56'23.0" | 41°02'49.8" | 19°10'47.1" |
| S12 | 38°58'35.3" | 21°51'56.2" | 40°32'56.9" | 19°56'06.9" | 41°03'31.0" | 19°08'56.4" |
| S13 | 38°58'44.8" | 21°51'35.2" | 40°48'14.7" | 19°40'21.3" |  |  |
| S14 | 39°10'13.0" | 21°29'42.2" | 40°48'21.1" | 19°40'36.6" |  |  |
| S15 | 39°09'40.7" | 21°30'25.1" | 40°47'38.0" | 19°35'42.2" |  |  |

**Table S1**: Raw data of Figure 1: Exact locations for each sample collected using Google earth.

A geological map of the Arabian Peninsula has been downloaded and opened using the software ArcGIS Pro (Version 2.8.3). From the spatial maps available on USGS website, rocks type in the area was determined using the legend provided by the USGS agency and the location of each sample taken was marked on the map as shown in **Fig. S1**.


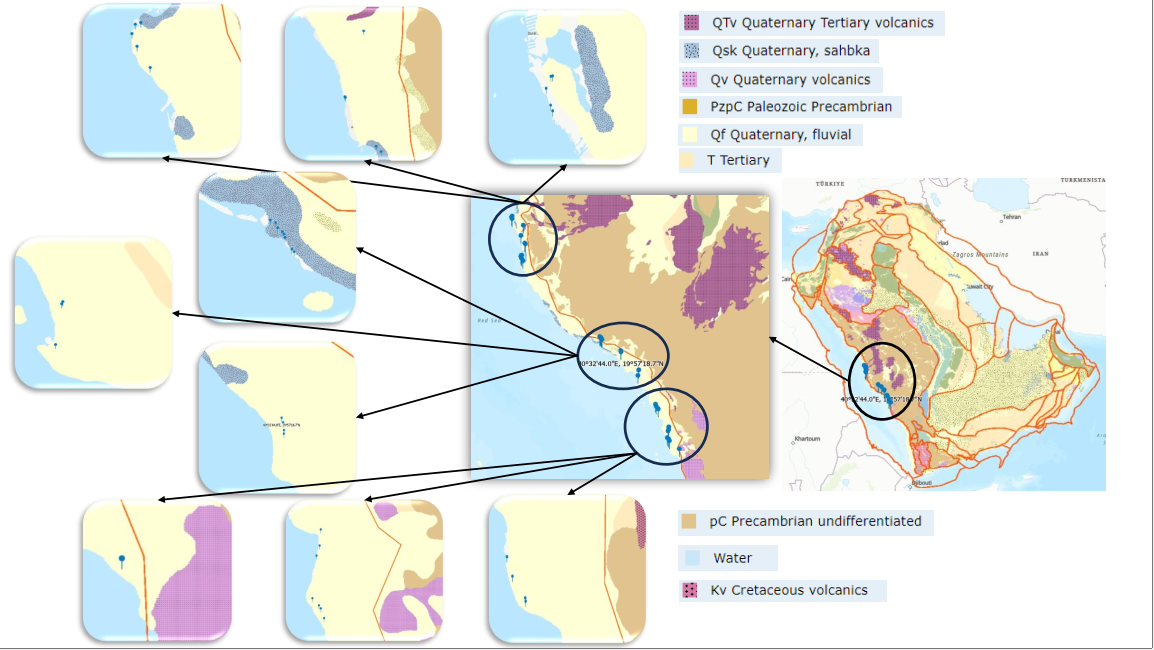


**2**

**3**

**1**

**Fig S1.** A spatial map showing the geology of the Arabian Peninsula, location of samples is highlighted with blue markers. **1**: Jeddah beach; **2**: Al Lith beach; and **3**: Al Qunfudhah beach

**Table S2:** Raw data of heavy metal concentrations (ppm) on Jeddah beach

| **Heavy metals concentrations (ppm)** | | | | | |
| --- | --- | --- | --- | --- | --- |
| **Fe** | **Pb** | **Cu** | **Mn** | **Zn** | **Cd** |
| 4622.04 | 15.56 | 19.06 | 56.12 | 35.6 | 0.82 |
| 748.99 | 7.92 | 9.71 | 12.88 | 56.33 | 1.2 |
| 917.14 | 15.86 | 6.79 | 14.61 | 489.73 | 2.9 |
| 2391.47 | 3.32 | 8.17 | 54.16 | 650.74 | 2.01 |
| 22613.63 | 12.54 | 5.53 | 56.66 | 48.43 | 1.46 |
| 9840.33 | 7.09 | 10.38 | 112.32 | 75.71 | 0.49 |
| 3875.77 | 19.58 | 10.38 | 52.38 | 48.95 | 2 |
| 10767.01 | 10.6 | 7.54 | 33.13 | 83.47 | 0.45 |
| 1674.69 | 8.58 | 14.43 | 56.57 | 74.39 | 1.3 |
| 1236.47 | 12.73 | 9.95 | 58.04 | 589.12 | 3.4 |
| 2713.9 | 5.83 | 6.95 | 50.33 | 497.2 | 2.76 |
| 4334.08 | 11.2 | 4.3 | 40.5 | 151.52 | 1.46 |
| 4093.34 | 15.3 | 31.83 | 20.89 | 79.83 | 2.57 |
| 6453 | 9.3 | 27.21 | 550.69 | 42.35 | 0.91 |
| 5379.08 | 6.4 | 4.26 | 95.62 | 37.3 | 2.08 |

**Table S3:** Raw data of heavy metal concentrations (ppm) on Al-Qunfudhah beach

| **Heavy metals concentrations (ppm)** | | | | | |
| --- | --- | --- | --- | --- | --- |
| **Fe** | **Pb** | **Cu** | **Mn** | **Zn** | **Cd** |
| 19007.67 | 0.86 | 16.36 | 472.31 | 94.66 | 4.94 |
| 11937.35 | 10.7 | 16.69 | 191.58 | 81.91 | 1.41 |
| 16564.34 | 17.41 | 12.92 | 286.16 | 81.38 | 2.91 |
| 22105.22 | 10.41 | 3.73 | 204.15 | 46.01 | 3.51 |
| 13324.84 | 2.02 | 11.85 | 197.88 | 51.53 | 2.56 |
| 12453.7 | 61.97 | 7.38 | 366.4 | 83.84 | 3.22 |
| 9082.17 | 19.67 | 12.51 | 303.07 | 83.75 | 2.05 |
| 3248.32 | 39.6 | 9.98 | 71.4 | 75.35 | 1.39 |
| 11417.25 | 27.28 | 13.6 | 231.82 | 54.76 | 2.91 |
| 15544.72 | 25.16 | 18.92 | 225.24 | 152.98 | 3.56 |
| 11961.71 | 16.3 | 7.09 | 341.56 | 50.3 | 1.97 |
| 16756.99 | 26.14 | 10.72 | 192.38 | 78.1 | 2.78 |
| 14852.08 | 0.16 | 16.7 | 182.69 | 68.25 | 3.97 |
| 14554.55 | 1.57 | 6.46 | 358.66 | 61.51 | 2.96 |
| 7439.06 | 13.68 | 16.7 | 182.32 | 69.89 | 2.36 |

**Table S4:** Raw data of heavy metal concentrations (ppm) on Al-Lith beach

| **Heavy metals concentrations (ppm)** | | | | | |
| --- | --- | --- | --- | --- | --- |
| **Fe** | **Pb** | **Cu** | **Mn** | **Zn** | **Cd** |
| 6690.6 | 19.45 | 69.51 | 149.9 | 139.43 | 2.63 |
| 12047.6 | 13.37 | 13.28 | 231.39 | 93.23 | 3.9 |
| 12012.64 | 14.768 | 18.087 | 124.04 | 137.59 | 3.227 |
| 22741.96 | 0.908 | 24.069 | 285.903 | 130.76 | 4.54 |
| 15040.36 | 10.325 | 14.015 | 209.488 | 90.393 | 3.765 |
| 20307.09 | 15.734 | 30.429 | 302.719 | 110.913 | 5.188 |
| 20544.9 | 24.18 | 22.34 | 197.68 | 87.02 | 4.69 |
| 19745.67 | 9.53 | 41.43 | 314.84 | 177.68 | 4.28 |
| 2372.07 | 9.45 | 13.53 | 38.09 | 76.02 | 2.63 |
| 2434.98 | 16.09 | 4.67 | 58.02 | 20.87 | 1.62 |
| 8753.78 | 8.4 | 15.26 | 105.4 | 45.94 | 2.54 |
| 2525.41 | 13.75 | 5.97 | 31.74 | 53.2 | 2.54 |
| 3050.92 | 13.62 | 11.43 | 60.76 | 33.35 | 2.47 |
